# Perioperative Cannabis Use in Bariatric Patients: A Review of Outcomes and Proposed Clinical Pathway for Management

**DOI:** 10.1007/s11695-024-07281-7

**Published:** 2024-05-21

**Authors:** Meghan H. Maceyko, Marc Neff, Jonathan Halevy, Marguerite Dunham

**Affiliations:** 1 Department of Surgery, Thomas Jefferson University Hospital, 1015 Walnut Street, Curtis Building, Suite 613, Philadelphia, PA 19107 USA; 2 Department of General Surgery, Jefferson Health New Jersey, Cherry Hill, NJ USA; 3 Department of Anesthesiology, Jefferson Health New Jersey, Cherry Hill, NJ USA; 4 Institute for Metabolic and Bariatric Surgery, Jefferson Health, Warminster, PA USA

**Keywords:** Cannabis, Bariatric, Marijuana, Complication

## Abstract

Legalization of marijuana has led to increased prevalence of medical and recreational cannabis use, underscoring the importance for anesthesiologists, surgeons, and perioperative physicians to understand the effects of this drug in patient care. Bariatric surgical patients represent a unique target population to understand peri-operative cannabis use and its effects as these patients undergo an extensive preoperative psychological and nutritional evaluation. Standardized guidelines on cannabis use in bariatric surgery are lacking and many clinicians remain uncertain on how to handle cannabis use in the peri-operative period. Here, we summarize the data on cannabis use in bariatric patients, specifically exploring anesthetic considerations, weight loss, complications, mortality, and psychiatric outcomes. We propose a clinical pathway to assist clinicians with perioperative decision making in bariatric patients who use marijuana.

## Introduction

In 2021, approximately 260,000 Americans underwent a weight loss operation [1]. Some studies report that up to 25% of patients do not achieve expected weight loss outcomes after bariatric surgery [2]. In order to optimize weight loss outcomes and minimize complication, a multidisciplinary approach is used as part of the pre-surgical preparation to address co-morbidities that may impede optimal surgical outcomes including nutritional, psychosocial, and medical evaluations.

Currently, the only contraindications to bariatric surgery are persistent alcohol and drug dependence, uncontrolled psychiatric illnesses, and cardiopulmonary diseases that would make the risk prohibitive. One such co-morbidity is substance use. The updated 2019 clinical practice guidelines for non-surgical support of patients undergoing bariatric procedures identify significant alcohol use and other substance use disorders as exclusion criteria for bariatric surgery [3]. However, these guidelines do not provide specific recommendations for the management of patients undergoing bariatric evaluation who report use of cannabis.

Marijuana is the most used recreational drug in the USA with about 10% of the population (26 million) reporting monthly use in 2017. It is estimated that about 1 in 10 of people who use marijuana will become addicted, with increased risk if use begins prior to the age of 18 (1 in 6 will become addicted) [4]. Legalization and decriminalization over the last decade have led to increased prevalence of both medical and recreational cannabis, underscoring the importance for anesthesiologists, surgeons, and perioperative physicians to understand the physiology and long-term outcomes of this drug in patient care. Given these ongoing changes, continued evaluation of the literature is critical.

Bariatric surgical patients represent a unique target population to understand peri-operative cannabis use and long-term effects as they undergo an extensive preoperative work up including psychological and nutritional evaluation. Patients with obesity have a high prevalence of substance use disorder history, which may include marijuana, and are routinely asked about their substance use history as alteration in gastrointestinal anatomy associated with bariatric surgery may lead to increased risk of substance use disorder [5]. It has been estimated that 6–8% of bariatric patients admit to using cannabis [6].

Standardized guidelines on cannabis use in bariatric surgery are lacking. Many clinicians are uncertain how to handle cannabis use given the varying legal status of medical and recreational use, limited research, difficulty quantifying use given multiple modes of delivery. In this review, we explore the effects of cannabis use on anesthetic considerations, post-operative weight loss, complications, mortality, and psychiatric outcomes in bariatric patients. Additionally, we propose a clinical pathway to assist clinicians in the peri-operative decision making when caring for bariatric patients who use cannabis.

## Relevant Terminology, Physiology, and Pharmacology

Clarification of terminology is important to assist physicians in their patient encounters as well as their understanding of the current literature with regards to cannabis. Cannabis itself includes all plant materials, components, and derivative products of the cannabis plant including but not limited to flowers, leaves, seeds, stalks, etc. Marijuana is a slang or colloquial term used interchangeably with cannabis in reference to plant strains with high Δ9-tetrahydrocannabinol (THC) content [7]. Cannabis use disorder (CUD) is defined by the DSM-V as the problematic pattern of use leading to impairment or distress symptoms within 12 months (e.g., inability to cut down, spending excess time to obtain or use, cravings, failure to fulfill major life obligations, tolerance, withdrawal) [8].

The two main chemicals derived from cannabis include cannabidiol (CBD) and Δ9-tetrahydrocannabinol (THC). THC is the primary cannabinoid in almost all varieties of cannabis. It is the main psychoactive agent and contributes to both the therapeutic and adverse effects. THC is a partial agonist at cannabinoid 1 (CB1) and cannabinoid 2 (CB2) receptors [9]. Agonism of the CB receptors results in changes in mood, memory processing, and motor control via neurotransmitter release (specifically acetylcholine, glutamate, and dopamine). This contributes to the analgesic and reward mechanisms within the nervous system. Inhalation of THC is the most common form of consumption. Inhalation leads to rapid absorption, increased bioavailability, and higher blood concentrations of THC. This contrasts with oral absorption which is undergoes hepatic metabolism resulting in lower bioavailability [9].

CBD has low affinity for the CB1 and CB2 receptors but rather interacts with additional receptors that regulate pain perception, such as serotonin 1A receptors. CBD is responsible for the potential analgesic, anti-epileptic, anxiolytic, and anti-inflammatory properties of cannabis. Like oral THC, oral CBD undergoes hepatic metabolism with oral intake (predominantly via cytochrome P450 enzymes). CBD has low toxicity; however side effects include somnolence, fatigue, appetite stimulation, and sleep changes [9].

## Perioperative Risks of Marijuana in Elective Surgery

With the expanding availability and legality of cannabis in the USA, peri-operative guidance for surgeons and anesthesiologists for patients reporting cannabis use is increasingly important. Cannabis is a drug with known effects on neurologic, cardiopulmonary, and gastrointestinal systems. Obesity also results in metabolic derangements and comorbidities that affect nearly every organ system of the body. Therefore, understanding the peri-operative risks of cannabis use is important in understanding the additional risk that cannabis asserts in the population of cannabis users undergoing bariatric surgery.

The cardiac effects of cannabis are dose-dependent. When used acutely in low doses, sympathetic nervous system activation leads to hypertension and tachycardia. When used chronically at high doses, there is risk of hypotension and bradycardia [8]. One study found that patients who use cannabis are at increased risk of peri-operative myocardial infarction [OR 1.88 (95% CI 1.31–2.69; *P* < 0.001)], potentially resulting from increased oxygen demand and hemodynamic derangements [10]. Patients diagnosed with obesity are at increased risk of arrythmias, thought to be secondary to cardiac remodeling over time. It is possible that the risk of arrythmia in a patient who is obese and using cannabis is amplified; however, this has not been studied. As a result of this data, anesthesia often recommends avoiding cannabinoids within 72 hours of surgery [7].

Additionally, there are alterations in pulmonary function and respiratory physiology caused by cannabis that may affect ventilation both intra-operatively and post-operatively. Smoking cannabis has been associated with a dose-dependent impairment of large airway function, lung hyperinflation, and airflow obstruction. Recommendations from the American Society of Regional Anesthesia and Pain Medicine (ASRA) suggest considering those who regularly smoke cannabis to be at similar risk for pulmonary complications to those who smoke tobacco [7]. Cannabis users in the general population have higher rates of pulmonary infection and impaired alveolar macrophage dysfunction; however, data in surgical patients is limited [11]. According to a study by Lundstrom et al. [12], obesity is associated with a 30% greater chance of difficult or failed intubation. In combination, these observations suggest that patients diagnosed with obesity who smoke cannabis are at even greater risk of perioperative pulmonary complications.

Symptoms of cannabis withdrawal typically occur within 24–72 hours of cessation and peak within the first week of cessation. The diagnostic criteria for cannabis withdrawal according to the DSM-V include three or more symptoms (irritability, anxiety, sleep disturbance, decreased appetite, restlessness, depressed mood, abdominal pain, fevers, headache, tremors, diaphoresis) causing significant distress [8]. Cannabis withdrawal may present similarly to common post-operative complications, such as anastomotic leak or pulmonary embolism. Therefore, it is important to maintain a high level of suspicion for other post-operative complications and test for them as indicated.

Components of cannabis interact with the cytochrome P450 (CYP) enzymes and may potentiate or attenuate the effects of important medications including warfarin, direct-acting oral anticoagulants, clopidogrel, as well as other antiarrhythmics, anticonvulsants, and anti-depressants [7]. Published case reports show cannabis may inhibit the metabolism of warfarin leading to increased plasma concentrations and bleeding risk [13]. Cannabidiol inhibits CYP2C19, an isoenzyme responsible for the transformation of clopidogrel to its active metabolite. This could result in subtherapeutic levels metabolite and possibly increased risk of stroke [14]. Larger studies are warranted to better understand the mechanisms behind the drug-drug interactions.

In 2023, the ASRA analyzed current literature and offered consensus recommendations graded A-D, based on the present level of evidence. Recommendations graded A or B offer a high certainty of substantial (A) or moderate (B) benefit to patients. Grade A recommendations included universal screening before surgery (including type of product used, last consumption, route, frequency of use), postponement of surgery in patients with altered mental status suggesting acute cannabis intoxication, and counseling frequent users of the negative effects on post-operative pain control [7]. Notably, moderate evidence exists to suggest that smoking cannabis may have deleterious effects on airway resistance and adverse respiratory events; however, no Grade A or B recommendations were made to suggest smoking cannabis should preclude patients from elective surgery. This data suggests all patients should be screened for cannabis use but an individualized approach should be employed for those undergoing general anesthesia who use cannabis. Special consideration may be had for patients who have concomitant cardiopulmonary disease.

## Marijuana Use and Perioperative Outcomes in Bariatric Patients

### Effects of Cannabis on Weight Loss Outcomes

It has been hypothesized that cannabis use would have a negative impact on weight loss following bariatric surgery because of the effects of THC and CBD on nervous system reward pathways and appetite stimulation. Interestingly, several studies suggest there is no difference in weight loss outcomes after bariatric surgery. Shockcor et al. report no difference in percent of total weight loss, percent excess weight loss, or BMI between marijuana users and non-users at 3 weeks, 3 months, 6 months, 1 year, and 2 years after bariatric surgery [15]. This study included just 71 bariatric patients and matched controls. Critics argued that maximum weight loss after bariatric surgery occurs greater than two years post-op. This was further examined in another study, where weight loss outcomes were measured up to five years post-op in 452 patients status post sleeve gastrectomy. There were no differences in excess weight loss noted between cannabis users and non-users both at early time points as well as 3 years and 5 years [16].

This contrasts with a more recent study of 765 bariatric patients which found that patients who reported any post-surgery use of cannabis had less percent excess weight loss and a higher likelihood of weight gain recurrence at 1-year follow-up. When looking at frequency of use, weekly cannabis use was associated with less percent excess weight loss and less percent total weight loss. Fifty-eight percent of weekly users has successful weight loss outcomes, defined by excess weight loss > 50%, compared to 73.3% of those without weekly use [OR 0.51 (0.30, 0.88), *P* = 0.02] [17]. This suggests that the frequency of cannabis use may be important in understanding the effects of cannabis on weight loss in this population.

Though the current data are limited, and further studies are warranted, it can be argued that the timeline (pre-operative, post-operative, etc.) and frequency of cannabis use may lead to differing post-operative weight loss outcomes in bariatric patients and should be regularly evaluated on pre-operative visits.

### Effects of Cannabis on Postoperative Complications and Mortality

Several studies have been done assessing rates of 30-day complications in bariatric patients using cannabis. One study found no difference in post-operative 30-day outcomes between cannabis users and non-users including readmission, reoperation, bleeding, and infection or length of stay [15]. A study by Janes et al. also reported no difference in 30-day outcomes (morbidity, mortality, abscess formation, anastomotic leak, bleeding, respiratory failure, renal failure, wound infection, venous thromboembolism, myocardial infarction) or health care utilization (length of stay, readmission, reoperation, Emergency Department visits) between cannabis users and non-users [18].

Interestingly, one study did note that patients undergoing sleeve gastrectomy who reported any marijuana use had a higher likelihood of reoperation within 30 days [19]. The authors stated that the reasoning was unclear and further research was warranted. Aside from weight loss, another goal of bariatric surgery is to improve the metabolic derangements associated with excess weight such as hypertension, diabetes, and hypercholesterolemia. Based on one study, there were no differences in discontinuation of insulin, oral hypoglycemic, antihypertensives, lipid lowering medications, or use of non-invasive positive pressure ventilation at 1 year post-op [18].

The diagnosis of Cannabis Use Disorder (CUD) may lead to variability in post-operative outcomes in the bariatric population. The re-admission rate after bariatric surgery is estimated to be 11.1% and CUD has been shown to be an independent predictor of readmission after bariatric surgery (aHR 3.37, 95% CI [1.05–10.01], *P* = 0.01) [20]. In the large study done by Shah et al., they describe that bariatric patients with CUD were more likely to experience a medical complication including myocardial infarction, cardiac arrest, venous thromboembolism, pulmonary embolism, respiratory arrest, pneumonia, sepsis, stroke, or urinary complications (4.8% vs 1.8%, *P* < 0.0001). Bariatric patients diagnosed with CUD were more likely to die (0.8% vs 0.2%, *P* = 0.002) and have a longer length of stay (3.7 vs 2.1 days, *P* < 0.001). These findings persisted on multivariate analysis [21].

## Marijuana Use and Psychiatric Outcomes in Bariatric Patients

Obesity is associated with psychological comorbidities such as depression, substance use disorder, and binge eating disorder [22]. Persons genetically predisposed to psychiatric disease, including depression, anxiety, and schizophrenia have an increased risk of mental illness with long term use of marijuana. It can be hypothesized that bariatric patients who use cannabis may have increased risk of comorbid psychiatric conditions.

A study by Vidot et al. showed that patients with increased marijuana use after weight loss surgery and/or recent marijuana use reported within 30 days of surgery had higher scores on eating disorder and food addiction scales post-operatively [23]. This was a retrospective study using standardized questionnaires including the Eating Disorders Examination Questionnaire (questions reflecting severity of psychopathology of eating disorders), the Disordered Eating Questionnaire (assessing emotional eating and night eating), and the Yale Food Addiction Scale (a validated questionnaire based on DSM-IV substance use disorder criteria). Patients who use marijuana after WLS had higher prevalence of late-night snacking, eating when bored or lonely, and were more likely to report loss of control and overeating. Another study found that bariatric patients who used cannabis were more likely to screen positive for major depressive disorder on the PHQ-8 survey (9.2% versus 5.7%, *P* = 0.0072) and scored overall significantly higher on the PHQ-8 (7.35 versus 3.62, *P* < 0.0001) [18]. The PHQ-8 survey is an 8 point depressive screening tool commonly used to evaluate the severity of depressive symptoms.

A study by Janes et al. showed that, compared with nonusers, bariatric cannabis users were more likely to have any co-morbid psychiatric disorder, more likely to be current smokers (6.7% versus 2%, *P* = 0.0003), and scored significantly higher on the alcohol use disorders identification test (AUDIT-C) (1.88 versus 1.11, *P* < 0.0001) at 1 year [18]. In a smaller study (*n* = 18), patients undergoing weight loss surgery had serotonin levels collected from duodenal tissue during surgery. Serotonin served as a biomarker for anxiety and depression as serotonin is a known modulator of anxiety, fear, and impulsivity. Cannabis users had higher tissue levels of serotonin than never users, suggesting that neurotransmitter dysregulation is present in bariatric patients who use cannabis and may contribute to addictive behavior and mood disorders [24].

Quantifying pre-operative marijuana use may predict post-operative use after bariatric surgery. In one study, 52% bariatric patients who used marijuana weekly pre-operatively reported daily use at 1 year post-operatively. Similarly, 42% of monthly users pre-op reported daily use at 1 year post-op [17]. This may suggest that patients who report pre-op use are at increased risk of more frequent use and may be at higher risk of developing cannabis use disorder. In summary, this data suggests that in bariatric patients who use cannabis, there is increased risk of depression, alcohol use, and disordered eating. This suggests that patients should undergo a thorough pre-operative screening of cannabis use and co-morbid psychiatric conditions to identify those who may benefit from close collaboration with psychologists and addiction specialists.

There is growing evidence that long-term, frequent cannabis users experience increased post-op pain and require more opioids than non-users [25]. In one study, 434 patients undergoing Roux-en-Y gastric bypass, sleeve gastrectomy, or gastric banding self-reported cannabis use pre-operatively. The results showed no difference in 30-day surgical site infections, readmissions, or ED visits however did demonstrate a statistically significant difference in total opioid use during hospitalization (47 mg in cannabis users vs. 37 mg in non-users, *P* = 0.0015) [26].

Finally, there is data to suggest that bariatric patients who use cannabis exhibit decreased post-operative appointment compliance at 6 months (38.4% vs. 54.8%, *P* = 0.046), 1 year (27.4% vs 41.1%, *P* = 0.081), and 2 years (12.3% vs 27.4%, *P* = 0.023) [15]. It is important to note that early post-operative visits with clinicians are imperative to ensure both dietary compliance and post-operative healing.

## Proposed Clinical Pathway for Bariatric Clinicians

**Fig. 1:**
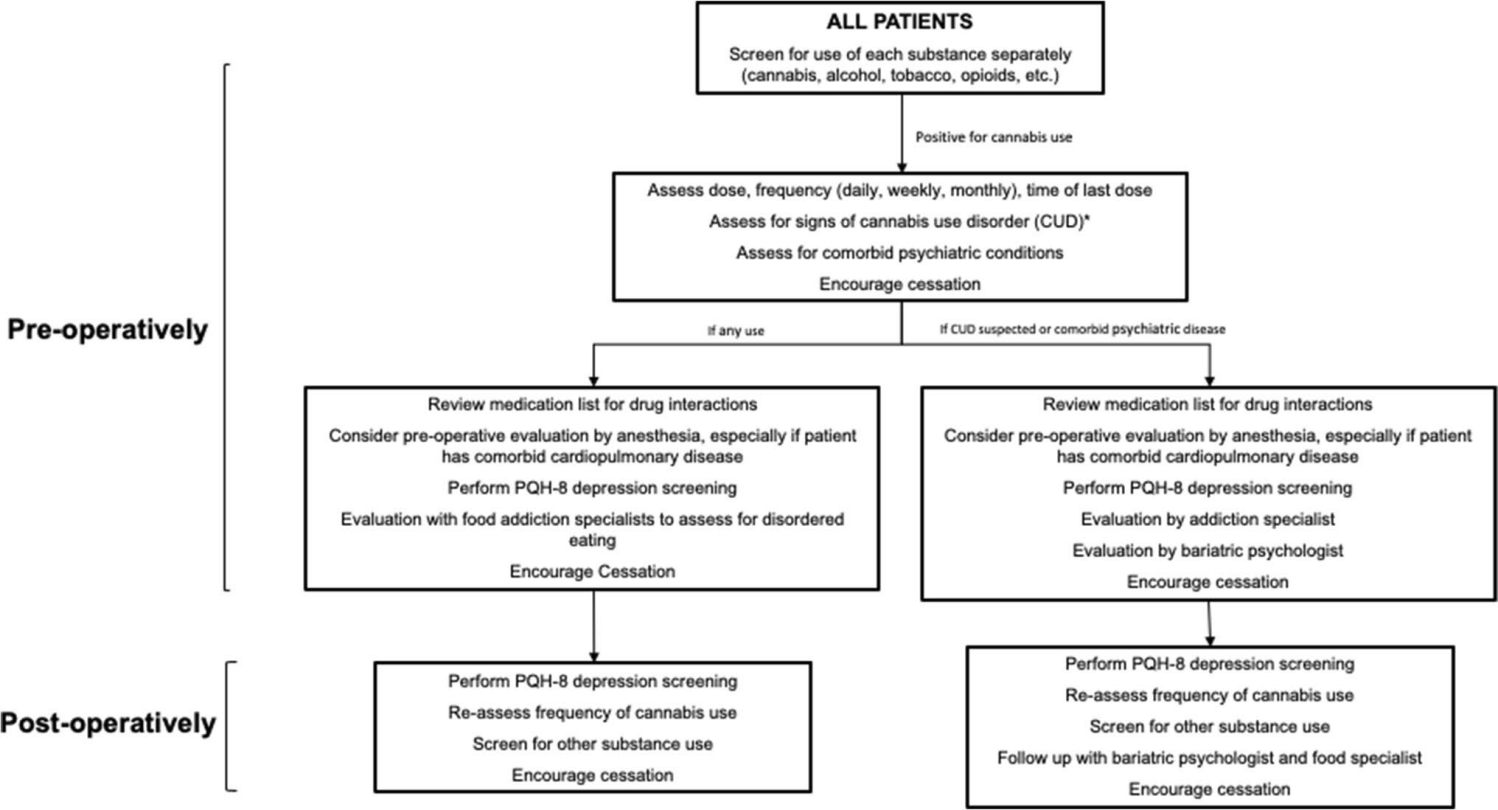
Proposed clinical pathway. *CUD as defined by the Diagnostic and Statistical Manual of Mental Disorders-V (DSM-V)

Many bariatric centers require a period of abstinence from alcohol, tobacco, and other substances before having surgery. However, little formal guidance and lack of consistency exists for bariatric providers with regards to the management of cannabis use before and after surgery. Several groups have recommended abstinence from cannabis ranging anywhere from 72 hours to 10 days prior to surgery; however, there is a lack of published data to recommend a specific duration. Here, we propose a standardized clinical assessment of cannabis use with recommended interventions for use in the bariatric population (Fig. 1). Preoperatively, all patients should be screened for substance use separately. For patients who reports cannabis use, dose, duration, frequency, and route of administration should be assessed. All patients who report cannabis use should undergo a review of medications to assess for drug-drug interactions. Given that cannabinoids can produce significant physiologic changes leading to peri-operative cardiac and pulmonary complications, close consultation with anesthesiology is recommended to mitigate these risks. Bariatric patients who use cannabis are at increased risk of depression post-operatively and should therefore be screened pre-op and post-op to monitor for early warning signs. Additionally, these patients may benefit from evaluation by a food addiction specialist to understand eating behaviors and work on pitfalls given their increased post-op risk of disordered eating and food addiction.

Special attention should be paid to patients who have concomitant psychiatric disease or are suspected of having cannabis use disorder. These patients may benefit from collaboration with specialists. Those with psychiatric conditions may benefit from additional meetings with a bariatric psychologist and addiction specialist pre-operatively and post-operatively. Post-operatively, patients with CUD should also be carefully assessed for additional substance use, such as alcohol.

Early identification of cannabis use and standardization in practice will help identify and treat those patients at risk for post-operative complications. By engaging high-risk patients early on, there are opportunities to encourage cessation and prevent the potential anesthetic, medical, and psychiatric complications.

## Conclusion

With the growing legalization of cannabis, there has been increasing research into the perioperative effects of cannabis in the bariatric population over the past decade. In this review, we summarize the current data on cannabis use in bariatric patients, specifically exploring anesthetic and medication interactions, post-op weight loss, complications, and mortality, and psychiatric outcomes in this population.

Based on the current data, the effects of cannabis use in bariatric patients may be better understood by stratifying frequency of use preoperatively. In studies where cannabis use is reported as a binary variable (ie users versus non-users), there appears to be no difference in weight loss outcomes, 30-day complications (bleeding, anastomotic leak, respiratory failure, myocardial infarction, etc.), or health care utilization. However, when cannabis use is classified by frequency of use (daily, weekly, monthly), there may be differences in weight loss outcomes, with less weight loss in more frequent users. Furthermore, the diagnosis of cannabis use disorder may carry increased risk of postoperative medical complications, re-admission, and even death. Although further studies are warranted, this data suggests that bariatric providers should regularly evaluate the frequency of cannabis use and assess for signs of problematic patterns of use to understand the true risk of postoperative complications and weight loss.

Multiple prior studies have demonstrated correlations between marijuana use and coexisting mental health disorders in patients, specifically finding an increase in incidence of mood and anxiety disorders among those who report regular marijuana use [27, 28]. The literature in the bariatric population appears similar. Bariatric patients who use cannabis are more likely to suffer from food addiction and participate in disordered eating post-op. Additionally, the studies show that cannabis users are more likely to report increased post-op cannabis use and concomitant alcohol use, and experience comorbid psychiatric disorders including depression. With the increased risk of substance use, disordered eating, and depression, bariatric patients should undergo a thorough pre-operative screening of cannabis use and co-morbid psychiatric conditions to identify those who may benefit from close collaboration with psychologists and addiction specialists. Those patients who use cannabis and also have significant psychiatric history may require even closer monitoring post-op to detect other substance use, depression, and disordered eating behavior. The higher rates of smoking, alcohol use, and depression in these patients suggest that patients who report marijuana use may benefit from extra support, such as additional therapy sessions or substance use counseling in the perioperative period.

In summary, a thorough understanding of the bariatric patient’s frequency of cannabis use both pre-op and post-op as well as comorbid psychiatric conditions may be important in recognizing patients who are at higher risk of suboptimal weight-loss, medical complications, substance use, and depression. We propose a recommended clinical pathway to help standardize the assessment of cannabis use and identify patients who may benefit from close collaboration with addiction specialists and psychologists.
